# MicroRNAs as Potential Signatures of Environmental Exposure or Effect: A Systematic Review

**DOI:** 10.1289/ehp.1408459

**Published:** 2015-01-16

**Authors:** Karen Vrijens, Valentina Bollati, Tim S. Nawrot

**Affiliations:** 1 Centre for Environmental Sciences, Hasselt University, Diepenbeek, Belgium; 2 Center of Molecular and Genetic Epidemiology, Department of Clinical Sciences and Community Health, Università degli Studi di Milano, Milan, Italy; 3 Department of Public Health and Primary Care, Environment and Health Unit, Leuven Univeristy, Leuven, Belgium

## Abstract

Background: The exposome encompasses all life-course environmental exposures from the prenatal period onward that influence health. MicroRNAs (miRNAs) are interesting entities within this concept as markers and causation of disease. MicroRNAs are short oligonucleotide sequences that can interact with several mRNA targets.

Objectives: We reviewed the current state of the field on the potential of using miRNAs as biomarkers for environmental exposure. We investigated miRNA signatures in response to all types of environmental exposure to which a human can be exposed, including cigarette smoke, air pollution, nanoparticles, and diverse chemicals; and we examined the health conditions for which the identified miRNAs have been reported (i.e., cardiovascular disease, cancer, and diabetes).

Methods: We searched the PubMed and ScienceDirect databases to identify relevant studies.

Results: For all exposures incorporated in this review, 27 miRNAs were differentially expressed in at least two independent studies. miRNAs that had expression alterations associated with smoking observed in multiple studies are miR-21, miR-34b, miR-125b, miR-146a, miR-223, and miR-340; and those miRNAs that were observed in multiple air pollution studies are miR-9, miR-10b, miR-21, miR-128, miR-143, miR-155, miR-222, miR-223, and miR-338. We found little overlap among *in vitro*, *in vivo*, and human studies between miRNAs and exposure. Here, we report on disease associations for those miRNAs identified in multiple studies on exposure.

Conclusions: miRNA changes may be sensitive indicators of the effects of acute and chronic environmental exposure. Therefore, miRNAs are valuable novel biomarkers for exposure. Further studies should elucidate the role of the mediation effect of miRNA between exposures and effect through all stages of life to provide a more accurate assessment of the consequences of miRNA changes.

Citation: Vrijens K, Bollati V, Nawrot TS. 2015. MicroRNAs as potential signatures of environmental exposure or effect: a systematic review. Environ Health Perspect 123:399–411; http://dx.doi.org/10.1289/ehp.1408459

## Introduction

Most common diseases result from the combined effect of genes and environmental factors and the interactions between them. Epigenetic effects and non-coding gene products have gained research focus over the last two decades because protein-coding genes cannot account for all observed genomic effects. Here we focus on microRNAs (miRNAs) as key regulators of health and disease. miRNAs are endogenous, single-stranded, short non-coding RNA sequences (~ 22 nucleotides) that regulate gene expression at the posttranscriptional level. Since the first discovery of miRNAs in *Caenorhabditis elegans* (Lee et al. 1993), hundreds of miRNAs in eukaryotes have been identified to influence physiological processes such as development, growth, differentiation, immune reaction, and adaptation to stress (van Rooij et al. 2007; Xiao et al. 2007). Diverse disease states, such as cancer and heart failure, are associated with distinct miRNA signatures, suggesting that specific miRNA programs are activated in pathophysiological processes (Calin et al. 2005).

Recent advances in molecular biology opened the opportunity for new approaches in population-based studies, in which exposures to a broad spectrum of environmental pollutants are evaluated in concert with biological systems, a concept proposed as the “exposome” (Wild 2005). From this viewpoint, miRNAs could potentially be novel biomarkers of exposure. For the purpose of this review, we focused on the response of miRNAs to environmental exposures.

**Figure 1:**
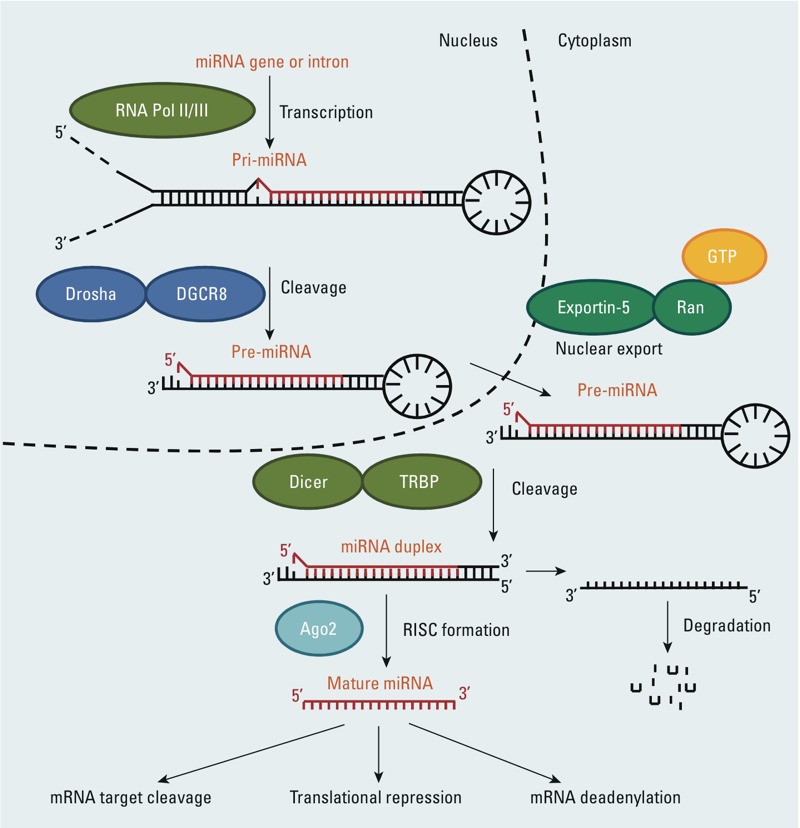
Overview of miRNA biogenesis. The canonical maturation of a miRNA includes the production of the primary miRNA transcript (pri-miRNA) by RNA polymerase II or III (Pol II/III) and cleavage of the pri-miRNA by the microprocessor complex Drosha–DGCR8 (Pasha) in the nucleus. The resulting precursor hairpin, the pre-miRNA, is exported from the nucleus by Exportin-5–Ran-GTP. In the cytoplasm, the RNase Dicer in complex with the double-stranded RNA-binding protein TRBP cleaves the pre-miRNA hairpin to its mature length. The functional strand of the mature miRNA is loaded together with Argonaute (Ago2) proteins into the RNA-induced silencing complex (RISC), where it guides RISC to silence target mRNAs through mRNA cleavage, translational repression, or deadenylation, whereas the passenger strand (black) is degraded.

*miRNA characteristics*. miRNA-mediated gene silencing is accomplished by base pairing of the 5´ region of miRNAs with the target mRNA sequence, leading to translational repression and/or mRNA degradation (Ambros 2004). miRNAs have been paradoxically shown to up-regulate gene expression by enhancing translation under specific conditions (Vasudevan et al. 2007). The effect of miRNA expression on gene expression is not linear, as multiple miRNAs may target the same mRNA, and the majority of mRNAs contain multiple binding sites for miRNAs, generating a highly complex regulatory network system (Saetrom et al. 2007). For details on miRNA synthesis, biogenesis, miRNA mechanism of action, see Figure 1 and reviews by Djuranovic et al. (2011) and Murchison and Hannon (2004).

*miRNA nomenclature*. miRNAs are named using the “miR” prefix and a unique identifying number (e.g., miR-1, miR-2). The identifying numbers are assigned sequentially, with identical miRNAs having the same number, regardless of organism. Paralogous sequences whose mature miRNAs differ at only one or two positions are given lettered suffixes: for example, miR-10a and miR-10b. Distinct hairpin loci that give rise to identical mature miRNAs have numbered suffixes (e.g., mir-281-1, mir-281-2). The mature sequences are designated “miR,” whereas the precursor hairpins are labeled “mir.” The -3p and -5p suffixes that sometimes appear within an miR name refer to the arm from which the mature sequence comes. For nomenclature guidelines, see Ambros et al. (2003).

*miRNA analysis techniques suitable for large epidemiological studies*. In recent years, miRNA expression changes following exposure to environmental toxicants, even before disease onset, have gained researchers’ interest. The measure of miRNAs in large epidemiological studies needs to be high throughput and sensitive enough to detect small changes in healthy subjects. At the same time, techniques need to be affordable in order to be conducted in large population studies. Moreover, given the complexity of phenomena induced by exposure but not fully explained by an effect on a single transcript, current research is going toward genome-wide techniques. Another challenge is tissue specificity of miRNAs: The availability of only noninvasive samples in epidemiological studies conducted on healthy populations limits our capability to investigate target tissues and opens important questions on the meaning of those markers in surrogate tissues. In epidemiological research, free and exosomal miRNAs in body fluids are interesting study objects because of their potential to serve as a proxy for tissue-specific miRNAs. A limitation of this approach is that these miRNAs differ between different body fluids, and their function is not clear. Although miRNAs hold promise as exposure biomarkers, recent studies have been primarily disease focused [reviewed by Etheridge et al. (2011)].

Genome-wide miRNA analysis can be achieved by amplification-based [real-time quantitative reverse-transcriptase polymerase chain reaction (qRT-PCR)], hybridization-based (microarrays), and sequencing-based [next-generation sequencing (NGS)] technologies. Method selection depends on the type of sample to be analyzed and the RNA preparation protocol used. qRT-PCR is considered the gold standard because of its sensitivity, specificity, accuracy, and simple protocols. qRT-PCR can evaluate candidate miRNA expression or array plates that include a large number of miRNAs in one reaction, to OpenArray® (Applied Biosystems, Life Technologies), which allows the simultaneous amplification of a very large panel of miRNAs using nanoscale volumes. In a recent review, Prokopec et al. (2013) compared qRT-PCR to different array-based platforms used to study mRNAs/miRNAs.

Several miRNA microarray chip platforms that are commercially available [e.g., Affymetrix GeneChip® 3.0 miRNA array (Affymetrix Inc.), Agilent Human miRNA Microarray system (Agilent Technologies), Exiqon miRCURY LNA™ microarray (Exiqon Inc.)] differ in probe design and detection stringency. The limitation of this microarray chip method is the availability and stringency of probes on the chip platform that pair with miRNAs of interest. Microarrays have the advantage of being easily correlated to mRNA expression data, thus providing functional information. Furthermore, unlike other current miRNA analysis techniques, microarrays allow fast analysis of miRNAs without an arbitrary preselection step. However, the large amount of data produced can generate false-positive results, and the time-consuming step of validation by qRT-PCR is almost necessary.

NGS strategies based on deep sequencing overcome some of the technical drawbacks of probe-based methodologies, especially the ability to detect only previously known sequences (Schulte et al. 2010). As miRNAs are sequenced directly, information about sequence variations or posttranscriptional RNA editing becomes available for further analysis. The newly developed Nanostring nCounter 27 (Nanostring Technologies Inc.) uses two sequence-specific capture probes to allow for discrimination between similar variants of a single miRNA. NGS technologies [e.g., Illumina/Solexa (Illumina Inc.), GA Roche/454 GS FLX Titanium (Roche Diagnostics Corp.), and ABI/SOLID (Applied Biosystems)] allow complete “miRnomes” to be sequenced and allow for the discovery of novel miRNAs and isoforms. Another benefit of NGS technology is that it can identify precursor and primary miRNAs as well as their mature forms. NGS will likely become the gold standard for miRNA analysis because of its ability to sequence short fragments in a high-throughput mode. The choice between these methods is a key factor in establishing the possibility of success of any epidemiological study. Each method has pros and cons and should be evaluated based on the specific research.

## Methods

**Figure 2:**
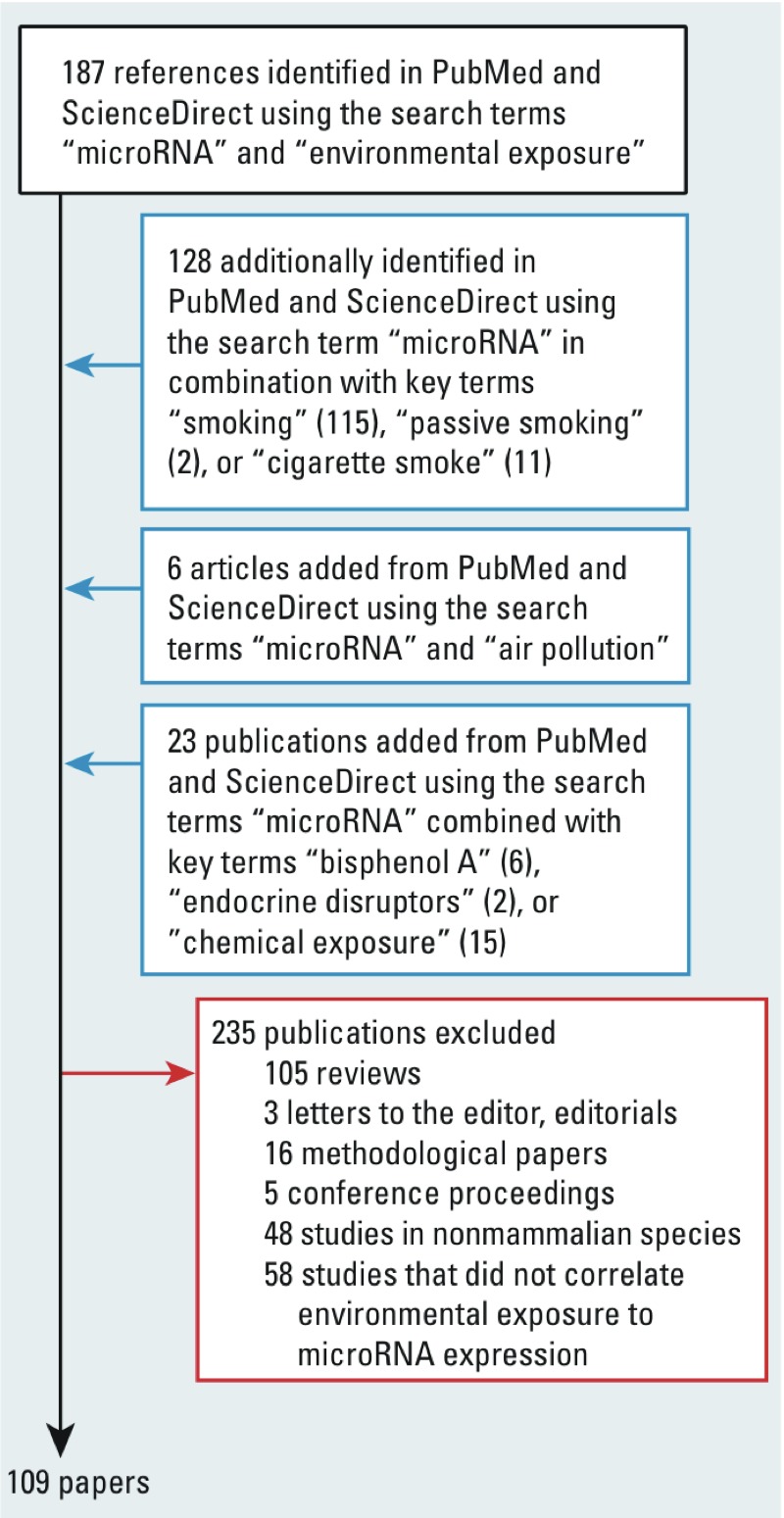
Flowchart of included studies.

*Search strategy and selection criteria*. To identify the articles relevant to this topic, we undertook a comprehensive search of the PubMed (http://www.ncbi.nlm.nih.gov/pubmed) and ScienceDirect (http://www.sciencedirect.com/) databases initially using “microRNA” and “environmental exposure” as key terms. We did additional searches in which we replaced “microRNA” with “mir,” “miRNA,” or “epigenetic changes” and we substituted “environmental exposure” with “smoking,” “passive smoking,” “cigarette smoke,” “air pollution,” “nanoparticle exposure,” “bisphenol A,” “endocrine disruptors,” or “chemical exposure” in every possible combination. We also considered review articles as well as references found in our literature search. We excluded articles not written in English. The PubMed search covered 1 January 1980 to 1 June 2014. Articles dealing only with the description of single nucleotide polymorphisms (SNPs) in miRNA genes were disregarded, as were those articles dealing only with the description of miRNAs in nonmammalian species. A flowchart detailing the search strategy is presented in Figure 2. For miRNAs differentially expressed in response to more than one personal or environmental exposure, we researched disease phenotypes correlated with them by searching each of these miRNAs on the Human microRNA Disease Database (HMDD; http://202.38.126.151/hmdd/mirna/md/) and the miR2Disease Base (http://www.mir2disease.org/). Results of these searches are presented in Table 1, including the direction of regulation (up or down) of the miRNA and the ensuing phenotype.

**Table 1:** miRNAs that are responsive to personal or environmental exposure and their roles in human disease.

miRNA	Regulated	Exposure	Diseases	Sources
Let-7e	Down	TCDD	HCC, lung, pituitary, and breast cancer, GEP tumors	Feitelson and Lee 2007; Qian et al. 2009; Rahman et al. 2009; Sakurai et al. 2012; Takamizawa et al. 2004
	Up	RDX	Heart failure, asthma	Polikepahad et al. 2010; Thum et al. 2007
Let-7g	Down	BPA, PM	Lung carcinoma, GEP tumors, breast cancer	Rahman et al. 2009; Sakurai et al. 2012
miR-9	Down	PM	Brain cancer, Huntingon’s disease	Ferretti et al. 2009; Lau and de Strooper 2010
	Up	Aluminum	Hodgkin lymphoma, breast cancer	Leucci et al. 2012; Ma et al. 2010
miR-10b	Down	Formaldehyde, PM	Gastric cancer	Kim K et al. 2011
miR-21	Down	Smoking	Diabetes type 2	Zampetaki et al. 2010
	Up	DEP, metal-rich PM	Breast cancer, glioblastoma, neo-intimal lesions, cardiac hypertrophy, atherosclerosis	Ji et al 2007; Raitoharju et al. 2011; van Rooij et al. 2007; Volinia et al. 2006
miR-26b	Down	DEP, BPA, PFOA	Schizophrenia, CRC, breast cancer	Earle et al. 2010; Liu et al. 2011; Perkins et al. 2007
miR-31	Down	DEP, TCDD	Medulloblastoma, T-cell leukemia	Ferretti et al. 2009; Yamagishi et al. 2012
miR-34b	Down	Smoking (2×)	CRC, pancreatic, mammary, ovarian, and renal cell carcinoma	Vogt et al. 2011
miR-92b	Down	Smoking, DDT	Medulloblastoma	Genovesi et al. 2011
miR-122	Down	Smoking	HCC	Bai et al. 2009
	Up	TCDD	Hepatitis C, renal cell carcinoma, male infertility, sepsis, hyperlipidemia	Gao et al. 2012; Henke et al. 2008; Wang C et al. 2011; Wang H et al. 2012; White et al. 2011
miR-125b	Down	Smoking (2×)	Breast cancer, head and neck cancer	Nakanishi et al. 2014; Zhang et al. 2011
	Up	Aluminum sulfate (2×)	Endometriosis, cardiac hypertrophy, Alzheimer’s disease	Busk and Cirera 2010; Lukiw and Alexandrov 2012; Ohlsson Teague et al. 2009
miR-135b	Down	DEP	Medulloblastoma	Lv et al. 2012
	Up	Smoking	CRC	Nagel et al. 2008
miR-142	Down	Formaldehyde	Heart failure	Voellenkle et al. 2010
	Up	Smoking	B-cell ALL	Ju et al. 2009
miR-143	Up	PM, ozone	Colon cancer	Zhang et al. 2013
miR-146a	Down	Smoking	Postpartum psychosis, type 2 diabetes	Weigelt et al. 2013; Zampetaki et al. 2010
	Up	BPA, aluminum sulfate (2×)	Alzheimer’s disease, Creutzfeldt-Jakob disease, atherosclerosis, leukemia, protection against myocardial injury	Lukiw and Alexandrov 2012; Lukiw et al. 2011; Raitoharju et al. 2011; Wang et al. 2013; Wang Y et al. 2010
miR-149	Up	BPA, DDT	Melanoma	Jin et al. 2011
miR-155	Down	PM	Hypertension	Xu et al. 2008
	Up	PM	Breast cancer, Hodgkin lymphoma, B-ALL	Chang et al. 2011; Kong et al. 2014; Palma et al. 2014
miR-181a	Down	Formaldehyde	Leukemia, glioma, NSCLC, breast cancer, metabolic syndrome, and CAD	Gao et al. 2010; Hulsmans et al. 2012; Marcucci et al. 2008; Ota et al. 2011; Shi et al. 2008
	Up	TCDD	Severe preeclampsia, male infertility	Hu et al. 2009; Wang C et al. 2011
miR-203	Down	Smoking, formaldehyde	Myeloma	Wong et al. 2011
miR-205	Up	Smoking (2×)	Heart failure, lung cancer	Thum et al 2007; Yanaihara et al. 2006
miR-206	Up	Smoking, RDX	Myocardial infarct, slows ALS progression, myotonic dystrophy	Gambardella et al. 2010; Shan et al. 2009; Williams et al. 2009
miR-222	Up	Metal-rich PM, BPA	Severe preeclampsia, thyroid carcinoma, prostate cancer, breast cancer	Hu et al. 2009; Miller et al. 2008; Pallante et al. 2006
miR-223	Down	Smoking	AML	Eyholzer et al. 2010
	Up	Smoking	Heart failure, atherosclerosis	Greco et al. 2012; Kin et al. 2012
miR-338-5p	Down	Formaldehyde	Melanoma	Caramuta et al. 2010
	Up	DEP	Oral carcinoma	Scapoli et al. 2010
miR-340	Down	Smoking	NA	NA
	Up	Smoking	Heart failure, breast cancer	Thum et al. 2007; Wu et al. 2011
miR-638	Up	BPA, DDT, arsenic	Lupus nephritis	Dai et al. 2009
miR-663	Up	BPA, DDT, arsenic	CTCL, nasopharyngeal carcinoma, burns	Liang et al. 2012; Ralfkiaer et al. 2011; Yi et al. 2012
Abbreviations: ACC, acute lymphocytic leukemia; ALS, amyotrophic lateral sclerosis; AML, acute myeloid leukemia; B-ALL, B-cell acute lymphocytic leukemia; BPA, bisphenol A; CAD, coronary artery disease; CRC, colorectal carcinoma; CTCL, cutaneous T-cell lymphoma; DDT, dichlorodiphenyltrichloroethane; DEP, diesel exhaust particles; GEP, gastroenteropancreatic; HCC, hepatocellular carcinoma; NA, not applicable; NSCLC, non-small cell lung carcinoma; PFOA, perfluorooctanoic acid; PM, particulate matter; RDX, hexahydro-1,3,5-trinitro-*s*-triazine; TCDD, 2,3,7,8-tetrachlorodibenzo-*p*-dioxin.				

## Results 

*Smoking-induced changes in miRNA expression*. The most studied environmental factor in relation to epigenetics is smoking; it was among the first factors shown to affect the miRNA machinery in humans (Spira et al. 2004). Results of *in vitro* studies concerning smoking and miRNAs are summarized in Table 2.

**Table 2:** *In vitro* studies on the effects of smoking on differential miRNA expression.

miRNA	miR function	Regulation	Tissue/cell type	Source
miR-15a	Tumor suppressor	Down	Primary bronchial epithelial cells	Schembri et al. 2009
miR-125b	Targets *p53*, stress response			
miR‑199b	Oncogene activation			
miR-218	Tumor suppressor			
miR-31	Apoptosis, tumor suppressor	Up	Normal and cancer lung cells	Xi et al. 2010
miR-21	Fatty acid synthesis, apoptosis	Up	Human squamous carcinoma cells	Zhang et al. 2014
miR-452	Targets *CDK1*	Down	Human alveolar macrophages	Graff et al. 2012

Izzotti et al. (2009) analyzed miRNA expression patterns in the lungs of mice exposed to passive cigarette smoke, and they established life-course–related miRNA expression changes by comparing miRNA expression in lungs from unexposed newborn, postweaning, and adult mice. These researchers observed developmental-stage–specific miRNA expression profiles in which miRNAs that were highly expressed in newborns tended to be less expressed in adult mice and vice versa, whereas miRNA expression in postweaning mice was intermediate (Izzotti et al. 2009). Results from *in vivo* studies concerning smoking and miRNAs are shown in Table 3.

**Table 3:** *In vivo* studies on the effects of smoking on differential miRNA expression.

miRNA	miR function	Regulation	Tissue/cell type	Source
miR-34b	p53 effector	Down	Mouse lung	Izzotti et al. 2011
miR-421	Targets *SMAD4*, polycomb gene *CBX7, ATM *			
miR-450b	No validated targets			
miR-466	No validated targets			
miR-469	Mouse miR not validated			
miR-135b	Inflammation, oxidative stress	Up	Mouse lung	Halappanavar et al. 2013
miR-206	Targets *SERP1, BDNF, FOXP1*	Up	Rat serum	Wu et al. 2013
miR-133b	Targets *LAG1, PTBP2*			
miR-20b	Hypoxia	Down	Mouse lung and plasma	Huang et al. 2012
miR-30e	Targets *UBC9, UBE21, MUC17*			
miR-125b	Targets *p53*, stress response			
miR-128	Apoptosis			
let-7a	Cell proliferation, angiogenesis	Down	Mouse lung	Izzotti et al. 2009
let-7b	Cell proliferation, angiogenesis			
let-7f	Cell proliferation, angiogenesis			
miR-26a	Transforming growth factor expression			
miR-30b	Cell adhesion, stress response			
miR-30c	Cell cycle, oncogene activation			
miR-34b	p53 effector			
miR-99b	Apoptosis			
miR-122a	Stress response			
miR-124a	Stress response, cell growth and differentiation			
miR-125a	Oncogene activation, ROS			
miR-125b	Targets *p53*, stress response			
miR-140	p53 effector			
miR-192	Oncogene activation			
miR-431	Protein repair, oncogene activation			
miR-92b	Tumor suppressomiR	Down	Mouse serum	Yuchuan et al. 2014
miR-668	Inflammation			
miR-700	Inflammation			
Let-7e	Apoptosis	Up	Mouse serum	Yuchuan et al. 2014
miR-19a	OncomiR			
miR-142	Immunology			
miR-191	OncomiR			
miR-350	Unknown			
Abbreviations: oncomiR, miR with oncogenic properties; ROS, reactive oxygen species; suppressomiR, tumor suppresor miR.				

Two studies reported a comparison between mRNA and miRNA whole genome expression patterns for smokers and nonsmokers (Schembri et al. 2009; Takahashi et al. 2013). Takahashi et al. (2013) reported that quitting smoking altered the plasma miRNA profiles to resemble those of nonsmokers. In addition, Let-7c and miR-150 could be of importance in the initiation of smoke-induced decline of lung function, because genes that were associated with lung function impairment in genome-wide association studies have been reported to be significantly enriched in binding sites for these miRNAs, namely *STAT3* (Qu et al. 2009) and *TNFR-II* (D’hulst et al. 2006).

The effect of *in utero* exposures on health during childhood and later in life is a growing area of research interest with major public health implications (Gluckman et al. 2008). An adaptive response in the fetus to *in utero* exposures can result in persistent changes into adulthood. miRNA expression levels in placenta can affect health later in life (Maccani et al. 2011). Studies on miRNA expression and human exposure at different stages of life (*in utero*, adult) are included in Table 4.

**Table 4:** Human studies on the effects of exposure to smoking on differential miRNA expression.

miRNA	miR function	Regulation	Tissue/cell type	Source
miR-16	p53, cell cycle, JAK/STAT signaling	Down	Placenta	Maccani et al. 2010
miR-21	Fatty acid synthesis, apoptosis			
miR-146a	Inflammation, NFκβ mediator			
miR-223	Immunology	Up	Maternal and cord blood	Herberth et al. 2013
miR-129	Cell cycle regulation, apoptosis	Down	Spermatozoa	Marczylo et al. 2012
miR-634	Inflammation			
miR-340	Cell migration and invasion	Up	Spermatozoa	Marczylo et al. 2012
miR-365	Targets *NKX2.1*			
miR-143	Cardiogenesis	Down	Gastric tissue	Stánitz et al. 2013
miR-21	Fatty acid biosynthesis, apoptosis	Up	Gastric tissue	Stánitz et al. 2013
Let-7c	Cell proliferation, angiogenesis	Down	Induced sputum	Van Pottelberge et al. 2011
miR-146a	Inflammation, NFκβ mediator			
miR-150	Hematopoeiesis			
miR-203	DNA damage response			
miR-340	Cell migration and invasion			
miR-443	Unknown			
miR-223	Immunology	Down	Plasma MV	Badrnya et al. 2014
miR-29b	Apoptosis	Up	Plasma MV	Badrnya et al. 2014
RNU6-2	Reference miR			
MV, microvesicles.				

**Figure 3:**
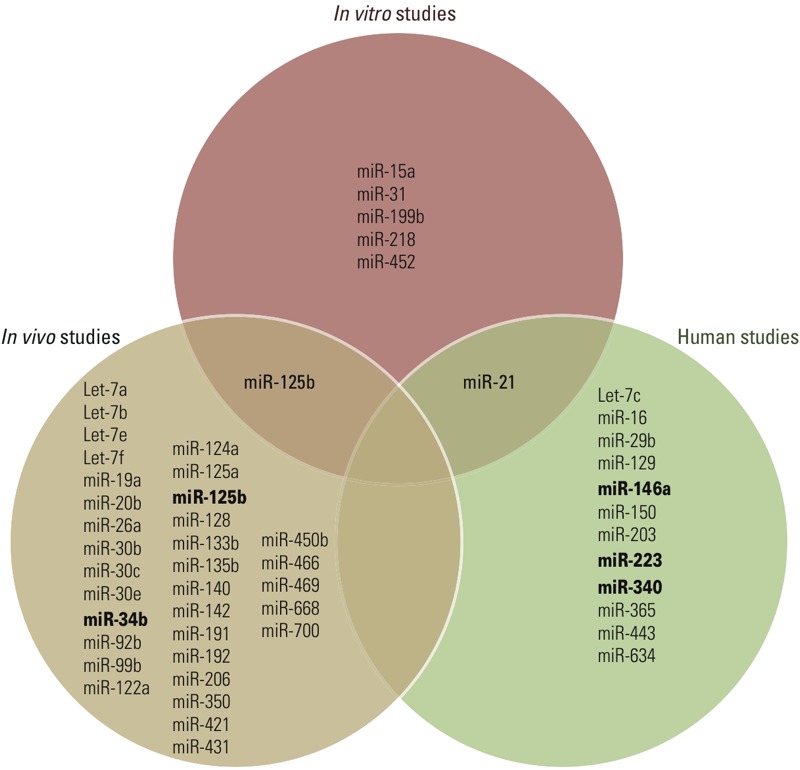
Venn diagram displaying common and distinct microRNAs associated with smoking in *in vitro*, *in vivo*, and human studies. miRNAs in bold type were identified in more than one study included in this meta-analysis.

Not surprisingly, miRNAs that are frequently observed to be down-regulated in response to smoking have also been identified as down-regulated in lung (Takamizawa et al. 2004), pancreatic (Vogt et al. 2011), and stomach (Rahman et al. 2009) cancer. Development of cardiovascular disease is associated with up-regulation of miR-206 (Shan et al. 2009), and this miRNA has significantly higher expression levels in smokers than in nonsmokers. Furthermore, two miRNAs that are frequently down-regulated in association with cigarette smoke (i.e., miR-21 and miR-146a) have lower expression levels in individuals with type 2 diabetes compared with healthy controls (Zampetaki et al. 2010). Therefore, these miRNAs could support the observation that smoking is an independent risk factor for type 2 diabetes (Cho et al. 2009). Human studies concerning smoking-induced changes of miRNA expression are summarized in Table 4. Figure 3 is a Venn diagram displaying the common and distinct miRNAs from *in vitro*, *in vivo*, and human studies on smoking-induced miRNA alterations. miR-125b and miR-21, identified in *in vivo* and human studies, respectively, were also reported in *in vitro* studies. Furthermore, several miRNAs were identified in multiple studies, such as miR-34b and miR-146a.

Table 1 summarizes miRNAs with altered expression in response to environmental and/or personal exposures reported in at least two independent studies, along with their known roles in disease etiology. miRNAs observed in association with either environmental or personal exposures are often associated with cancer; in particular, breast and lung cancer and leukemia have been frequently reported (Table 1). Furthermore, many aberrations in the cardiovascular system have been reported, such as hypertension, heart failure, myocardial infarct, and atherosclerosis. Exposures such as air pollution and smoking can cause cardiovascular disease and cancer (Pope et al. 2011); however, the data shown in Table 1 indicate that the listed miRNAs play a causative role in disease etiology, rather than being merely a marker of exposure.

*Air pollution exposure and miRNA expression*. Particulate matter (PM) is a complex mixture of small particles and liquid droplets. Particle pollution is made up of a number of components, including acids, organic chemicals, metals, and soil or dust particles. The size of particles is directly linked to their potential to cause health problems (Brunekreef and Holgate 2002). Although the clinical effects of PM exposure are obvious, the underlying mechanism of disease initiation and progression is less well understood. miRNAs play a pivotal role in maintaining healthy lungs (Nana-Sinkam et al. 2009). Because the lungs are an important target site for PM, we suggest that miRNAs could underlie the observed health effects of PM exposure. *In vitro* studies on air pollution and miRNAs are summarized in Table 5.

**Table 5:** *In vitro* studies on air pollution–induced changes in miRNA expression.

miRNA	miR function	Regulation	Tissue/cell type	Pollutant	Source
miR-26b	Wnt, p53, autophagy, TGF-β	Down	Primary human bronchial epithelial cells	10 μg/cm^2^ DEP	Jardim et al. 2009
miR-27a	Apoptosis, ERα				
miR-31	Apoptosis, tumor supressor				
miR-96	Several unrelated targets				
miR-135b	Inflammation, oxidative stress				
miR-374a	Targets *DICER*, *ATM*				
miR-513c	No validated targets	Up	Primary human bronchial epithelial cells	10 μg/cm^2^ DEP	Jardim et al. 2009
miR-513b	No validated targets				
miR-513a-5p	Targets *CD274*, immunology				
miR-923	Fragment of 28S RNA				
miR-494	Targets *PTEN*				
miR-338-5p	ABC transporters, endocytosis				
miR-10b	Angiogenesis	Down	Human A549 lung carcinoma cell line	1 ppm CH_2_O	Rager et al. 2011
miR-181a	Apoptosis, oncomiR				
miR-330	Targets *E2F1, VEGFa, NTRK3*				
miR-338-5p	ABC transporters, endocytosis				
miR-375	Immunology	Up	Human bronchial epithelial cells	3 μg/cm^2^ DEP	Bleck et al. 2013
miR-149	Immunology	Down	Monkey airway epithelial cells	Ozone	Clay et al. 2014
miR-128	Apoptosis	Up	Human A549 lung carcinoma cell line	PM_10_	Motta et al. 2013
Abbreviations: CH_2_O, formaldehyde; DEP, diesel exhaust particles; OncomiR, miR with oncogenic properties.					

In a cohort study of steel plant workers, Bollati et al. (2010) examined the effect of PM exposure on miRNA expression. Blood samples were collected at the beginning of the working week (“preexposure”) and at the end of the working week (“postexposure”). PM mass and metal components measured in the plant were correlated with miRNA expression analyses of blood samples. Urinary 8-hydroxy-2´-deoxyguanosine (8-OH-dG) levels were measured as a readout of oxidative stress. Both miR-222 and miR-21 were significantly increased in post- versus preexposure samples, and only miR-21 expression levels were positively correlated with 8-OH-dG (Bollati et al. 2010). Oxidative stress has been reported to induce miR-21 expression (Cheng et al. 2009); thus, the association between 8-OH-dG and miR-21 might simply reflect the response of miR-21 to production of reactive oxygen species (ROS) in the blood due to the PM-induced increase in oxidative stress (Bollati et al. 2010) (Table 6).

**Table 6:** Human studies on air pollution–induced changes in miRNA expression.

miRNA	miR function	Regulation	Tissue/cell type	Pollutant	Source
miR-21	Fatty acid synthesis, apoptosis	Up	Peripheral blood	300 μg PM_2.5_/m^3^ DEP	Yamamoto et al. 2013
miR-30e	Targets *UBC9, MUC17*				
miR-144	Targets *Klfd, FGG, PLAG1*				
miR-215	Cell cycle, *p53* activation				
miR-21	Fatty acid synthesis, apoptosis	Up	Blood leukocytes	Metal-rich PM	Bollati et al. 2010
miR-222	Cell cycle regulation				
miR-375	Immunology	Up	Bronchial epithelial cells	3 μg/cm^2^ DEP	Bleck et al. 2013
miR-34a	Cardiogenesis	Up	Gastric tissue	Urban living	Stánitz et al. 2013
miR-143	Cardiogenesis				
miR-10b	Angiogenesis	Up	Spermatozoa	Metal-rich PM	Li et al. 2012a
miR-33b	Lipid metabolism				
miR-106a	OncomiR				
miR-155	Inflammation				
miR-183	OncomiR				
miR-205	OncomiR				
miR‑208a	Cardiac hypertrophy				
miR-222	Cell cycle regulation				
miR-223	Immunology				
Let-7d	Proliferation, angiogenesis	Down	Spermatozoa	Metal-rich PM	Li et al. 2012a
miR-363	DNA damage response				
miR-25	DNA damage response	Up	Induced sputum	Ozone	Fry et al. 2014
miR-132	Angiogenesis				
miR-143	Cardiogenesis				
miR-145	Tumor suppressor				
miR-199a	Oncogene activation				
miR-199b	Oncogene activation				
miR-222	Cell cycle regulation				
miR-223	Immunology				
miR-424	Angiogenesis				
miR-582	Antiapoptosis				
miR-1	Apoptosis	Down	Leukocytes	PM_2.5_, black carbon, organic carbon, sulfate	Fossati et al. 2014
miR-9	Neuronal differentiation				
miR-21	Fatty acid synthesis, apoptosis				
miR-126	Angiogenesis				
miR-135a	Inflammation				
miR-146a	Inflammation, NFκβ mediator				
miR-155	Inflammation				
miR-222	Cell cycle regulation				
miR-128	Apoptosis	Up	Plasma MV	PM_10_	Motta et al. 2013
Abbreviations: DEP, diesel exhaust particles; MV, microvesicles; OncomiR, miR with oncogenic properties; PM_2.5_, particulate matter ≤ 2.5 μm in aerodynamic diameter.					

The cardiovascular anomalies observed in association with air pollution exposure have often been attributed to the generation of oxidative stress (Miller et al. 2012). MiR-21 is up-regulated in response to diesel exhaust particles and metal-rich PM (Bollati et al. 2010; Bourdon et al. 2012) and is highly expressed in the cardiovascular system, where it plays an important role in vascular cell proliferation and apoptosis and disease [reviewed by Cheng and Zhang (2010)]. Therefore, miR-21 expression could be an important mechanistic link explaining the association between air pollution exposure and cardiovascular disease.

**Figure 4:**
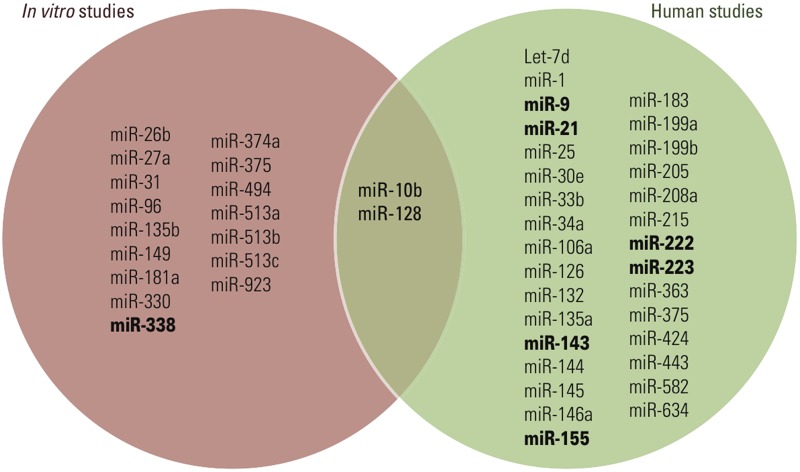
Venn diagram displaying common and distinct microRNAs associated with air pollution exposure in *in vitro* and human studies. miRNAs in bold type were identified in more than one study included in this meta-analysis.

Levänen et al. (2013) observed distinct miRNA expression profiles in patients with asthma compared with controls after subway exposure. Current epidemiological studies have identified the first miRNAs associated with air pollution exposure, and provide a list of putative biomarkers. Table 6 summarizes the human studies on air pollution and miRNAs. A Venn diagram displays the common and distinct miRNAs from *in vitro* and human studies on air pollution–induced miRNA alterations (Figure 4). The only miRNAs identified in both *in vitro* and human studies in association with air pollution exposure are miR-10b and miR-128. Furthermore, miRNAs -9, -21, -143, -155, -222, -223, and -338 were identified in at least two independent studies on air pollution and miRNA.

*Nanoparticles*. Nanoparticles are emitted from natural and anthropogenic sources and are produced via nanotechnology. Fast propagation of nanotechnologies into different industries and consumer products is causing exponential growth of nanomaterial production. Hence, increasing amounts of nanoparticles reach occupational settings and the indoor and outdoor environments, thus representing a potentially serious hazard to human health (Castranova 2011; Nel et al. 2006). Nanoparticles are also able to cross cell membranes, and their interactions with biological systems are relatively unknown (Holsapple et al. 2005). Table 7 includes the studies on nanoparticle-induced changes in miRNA expression, all of which were performed in animal models.

**Table 7:** Studies on nanoparticle-induced changes in miRNA expression.

miRNA	miR function	Regulation	Pollutant	Source
miR-21	Fatty acid synthesis, apoptosis	Up	0.268 or 0.162 mg carbon black NP	Bourdon et al. 2012
miR-135b	Inflammation, oxidative stress			
miR-146	Inflammation, NFκβ mediator			
miR-122	Stress response	Up	70 nm in silica NP	Nagano et al. 2013
miR-192	Oncogene activation			
Let-7a	Cell proliferation, angiogenesis	Up	100 nm gold NP	Balansky et al. 2013
miR-183	OncomiR			
Abbreviations: NP, nanoparticles; oncomiR, miR with oncogenic properties.				

*Chemical exposure-induced changes in miRNA*. Formaldehyde. Formaldehyde is an air toxic present in the atmosphere due to emission from anthropogenic and biogenic sources. Ninety-five percent of inhaled formaldehyde is absorbed within the respiratory tract (Overton et al. 2001). Formaldehyde has been reported to change gene expression patterns in nasal and lung cells (Kim et al. 2002; Li et al. 2007). The miRNAs reported to be down-regulated in association with formaldehyde exposure have been reported to be involved in the development of diverse tumors (e.g., breast and gastrointestinal cancer, melanoma) as well as heart failure (Table 1). Given the capability of formaldehyde to pass deep into lung tissue and enter systemic circulation, the link with cardiovascular disease and cancer has been widely discussed [reviewed by Kim KH et al. (2011)]. Interestingly, miR-181a, one of the miRNAs down-regulated after formaldehyde exposure, was reported to affect the DNA damage response in breast cancer, enabling the identification of aggressive breast tumors based on increased miR-181a expression (Bisso et al. 2013).

Endocrine disruptors. Organochlorine pesticides and plasticizing agents are ubiquitous environmental endocrine-disrupting compounds that impact human health (Rubin 2011). Bisphenol A (BPA) is an industrial plasticizer often used as a coating in food cans and in plastic bottles (Kang et al. 2006). Dichlorodiphenyltrichloroethane (DDT) is a well-known organochlorine pesticide. Because DDT is very persistent in the environment, accumulates in fatty tissues, and can travel long distances in the upper atmosphere, residues from historical use remain a current threat to human health.

DDT and BPA have been reported to interfere with endogenous estrogens and thyroid hormone, leading to aberrations of the reproductive, immune, and central nervous systems (Chevrier et al. 2013; Liu et al. 2013). DDT (Waliszewski et al. 2001) and BPA (Takahashi and Oishi 2000) cross the placental barrier and can induce *in utero* effects that could lead to detrimental effects later in life.

Soto et al. (2013) reported that prenatal exposure to BPA can alter mammary development and lead to breast cancer in humans. From a clinical perspective, it is interesting that decreased expression of *let-7f* has been associated with increased breast cancer risk (Sakurai et al. 2012), and treatment of MCF-7 breast cancer cells with BPA resulted in reduced *let-7f* expression (Tilghman et al. 2012). Furthermore, miR-146a has been proposed to induce an Alzheimer’s disease pathway (Jiang et al. 2013) and is up-regulated after BPA exposure (Table 1). Therefore, the neurodegenerative consequences of BPA exposure could at least partially be attributed to miR-146a. *In vitro* studies could provide researchers with interesting miRNAs that have potential to be used as biomarkers for chemical exposure.

Polychlorinated biphenyls (PCBs) were widely used organic chemicals until their production was banned because of environmental concerns (Porta and Zumeta 2002). PCBs are stable compounds that bioaccumulate in fatty tissues (Steele et al. 1986); they have been reported to cause systemic changes in gene expression (Ceccatelli et al. 2006), suggesting that miRNA regulation could be involved in this process. Tsukimori et al. (2008) reported an association between maternal PCB exposure and fetal toxicity, impaired fetal growth, and pregnancy loss.

2,3,7,8-Tetrachlorodibenzo-*p*-dioxin (TCDD) has been reported to adversely affect the immune system in rats (Faith and Luster 1979). In addition, Camacho et al. (2004) reported that TCDD exposure of pregnant mice affected the immune system of fetuses by suppressing T-cell function. Given the regulatory role miRNAs play in the immune system (Contreras and Rao 2012), it can be expected that miRNAs are important in regulating the detrimental health effects observed after exposure to TCDD and PCBs.

**Figure 5:**
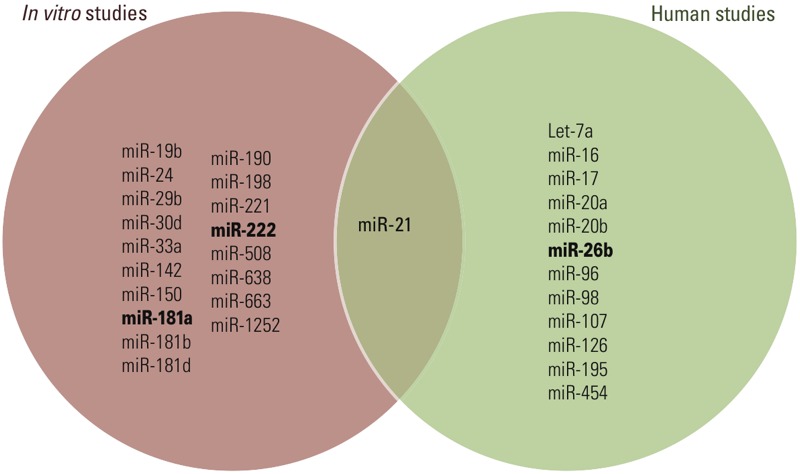
Venn diagram displaying common and distinct microRNAs associated with arsenic exposure in *in vitro* and human studies. miRNAs in bold type were identified in more than one study included in this meta-analysis.

Arsenic. Environmental exposure to arsenic, especially to trivalent inorganic arsenic (As^3+^), is a health concern given the high concentrations present in groundwater across the world (Fendorf et al. 2010). Exposure to arsenic has been associated with increased risk of cancer due to genomic instability (Dulout et al. 1996), and long-term arsenic exposure has been observed to induce peripheral vascular injury (Tseng 2008). A Venn diagram showing the common and distinct miRNAs from *in vitro* and human studies on arsenic-induced miRNA alterations is presented in Figure 5. Only miRNA-21 was associated with arsenic exposure in *in vitro* model systems and in human studies. Three miRNAs were identified by at least two independent studies on arsenic exposure and miRNA expression, namely, miR-26b, miR-181a, and miR-222.

Aluminum sulfates. Aluminum is the most widely distributed metal in the environment and is extensively used in daily life. Chronic exposure of animals to aluminum is associated with behavioral and neuropathological changes (Fulgenzi et al. 2014). Epidemiological studies have shown poor performance in cognitive tests and a higher abundance of neurological symptoms in workers occupationally exposed to aluminum (Kumar and Gill 2009).

Hexahydro-1,3,5-trinitro-s-triazine (RDX). The polynitramine explosive RDX is a heavily used second-generation high explosive, and its use can result in the contamination of soils, sediments, and water (Davis et al. 2004). RDX exposure has been reported to be toxic to the neural and immune systems and to increase tumor incidence in several cancers (Garcia-Reyero et al. 2011; Sweeney et al. 2012).

Diethylstilbestrol (DES). The synthetic estrogen DES was prescribed to pregnant women from the 1940s to the 1960s in order to prevent miscarriages; however, DES was later reported to be responsible for increasing breast cancer in the mothers and gynecologic tumor incidence in their exposed daughters (Greenberg et al. 1984; Mittendorf 1995).

Perfluorooctanoic acid (PFOA). Perfluoroalkyl chemicals (PFCs) are highly stable and widely used in industrialized countries. PFCs are both lipophobic and hydrophobic; thus, after absorption they will bind to proteins in serum and liver rather than accumulate in lipids. PFOA is one of the most commonly used PFCs.

The studies we reviewed on chemical-induced changes in miRNA expression are summarized in Tables 8–10 by type of study: *in vitro* (Table 8), *in vivo* (Table 9), and human (Table 10) studies.

**Table 8:** *In vitro* studies on chemically induced changes in miRNA expression.

miRNA	miR function	Regulation	Tissue/cell type	Chemical	Source
let-7g	Cell proliferation, angiogenesis	Down	MCF-7 cells	BPA	Tilghman et al. 2012
let-7f	Cell proliferation, angiogenesis				
miR-21	Fatty acid biosynthesis, apoptosis				
miR-26b	Wnt*, *p53*,* autophagy, TGF*-*β				
miR-342-3p	Tumor suppressomiR				
miR-15b	Tumor suppressor targeting *BCL2*	Down	MCF-7 cells	BPA, DDT	Tilghman et al. 2012
miR-222	Cell cycle regulation	Up	MCF-7 cells	BPA	Tilghman et al. 2012
miR-638	No known function	Up	MCF-7 cells	BPA, DDT	Tilghman et al. 2012
miR-663	Immunology, oxidative stress	Down	MCF-7 cells	DDT	Tilghman et al. 2012
miR-1915	No known function				
miR-27b	Angiogenesis				
miR-92a	Tumor supressomiR				
miR-92b	Tumor supressomiR				
miR-1308	No known function	Up	MCF-7 cells	DDT	Tilghman et al. 2012
miR-146a	Inflammation, NFκβ mediator	Up	Human placental cell lines	BPA	Avissar-Whiting et al. 2010
miR-150	Hematopoeiesis	Down	Jurkat T cell line	Arsenic	Sturchio et al. 2014
miR-30d	Autophagy	Up	Jurkat T cell line	Arsenic	Sturchio et al. 2014
miR-142	Immunology				
miR-181a	Apoptosis, oncomiR				
miR-221	DNA damage response				
miR-222	Cell cycle regulation				
miR-638	No known function				
miR-663	Immunology, oxidative stress				
miR-190	OncomiR	Up	Human bronchial epithelial cells	Arsenic	Beezhold et al. 2011
miR-19b	OncomiR	Up	HUVEC cells	Arsenic	Li et al. 2012b
miR-21	Fatty acid biosynthesis, apoptosis				
miR-24	OncomiR				
miR-29b	Apoptosis				
miR-33a	Lipid metabolism				
miR-198	Cell proliferation				
miR-508-5p	Cell invasion and migration				
miR-1252	No known function				
miR-181a	Apoptosis, oncomiR	Up	HepG2 cells	PAH	Song et al. 2013
miR-181b	Apoptosis, oncomiR				
miR-181d	Apoptosis, oncomiR				
Abbreviations: BPA, bisphenol A; DDT, dichlorodiphenyltrichloroethane; OncomiR, miR with oncogenic properties; PAH, polycyclic aromatic hydrocarbon; tumor suppressomiR, tumor suppressor miR.					

**Table 9:** *In vivo* studies on chemically induced changes in miRNA expression.

miRNA	miR function	Regulation	Tissue/cell type	Chemical	Source
let-7e	Apoptosis	Down	Fetal mouse thymocytes	TCDD	Singh et al. 2012
miR-18b	Apoptosis				
miR-23a	Apoptosis				
miR-23b	Apoptosis				
miR-27a	Apoptosis, ERα				
miR-28	Apoptosis				
miR-29a	Apoptosis				
miR-31	Apoptosis, tumor supressomiR				
miR-98	Apoptosis				
miR-101b	Apoptosis				
miR-181c	Apoptosis, oncomiR				
miR-182	Apoptosis				
miR-200a	Apoptosis, cell cycle, MAPK				
miR-23	Apoptosis				
miR-290	Apoptosis				
miR-335	Apoptosis				
miR-491	Apoptosis, targets *BCL-XL*				
miR-122	Stress response	Up	Fetal mouse thymocytes	TCDD	Singh et al. 2012
miR-181a	OncomiR				
miR-125b	Targets *p53*, stress response	Up	Monkey nasal epithelium	Formaldehyde	Rager et al. 2013
miR-152	Tumor suppressor, methylation				
miR-219	NMDA receptor signaling				
miR-532	Unknown				
miR-22	PTEN*, *AKT signaling	Down	Monkey nasal epithelium	Formaldehyde	Rager et al. 2013
miR-26b	Wnt, p53, autophagy, TGF-β				
miR-29a	Apoptosis				
miR-140	p53 effector				
miR-142	Immunology				
miR-145	Tumor suppressor, stem cell different				
miR-203	DNA damage response				
miR-374a	Targets *DICER, ATM*				
miR-520f	Unknown				
miR-27a	Apoptosis, ERα	Down	Mouse brain and liver	RDX	Zhang and Pan 2009
miR-200c	Apoptosis				
let7-e	Apoptosis				
miR-206	Targets *SERP1, BDNF, FOXP1*				
miR-451	Targets *PI3K*/*AKT*	Down	Rat liver	PFOS	Wang et al. 2014
miR-23a	Apoptosis	Up	Rat liver	PFOS	Wang et al. 2014
miR-25	DNA damage response				
miR-125a	Oncogene activation, ROS				
miR-133a	Smooth muscle differentiation				
miR-133b	Targets *LAG1* and *PTBP2*				
miR-206	Targets *SERP1, BDNF, FOXP1*				
miR-494	Targets *PTEN*				
miR-542	DNA damage response				
Abbreviations: OncomiR, miR with oncogenic properties; PFOS, perfluorooctane sulfonate; RDX, hexahydro-1,3,5-trinitro-*s*-triazine; TCDD, 2,3,7,8-tetrachlorodibenzo-*p*-dioxin.					

**Table 10:** Human studies on chemically induced changes in miRNA expression.

miRNA	miR function	Regulation	Tissue/cell type	Chemical	Source
miR-191	OncomiR	Up	Peripheral blood	PCB-169	Guida et al. 2013
miR-146a	Inflammation, NFκβ mediator	Up	Fetal brain cells	Aluminum	Pogue et al. 2009
miR-9	Neuronal differentiation				
miR-125b	Targets *p53*, stress response	Up	Fetal brain cells	Aluminum	Lukiw and Pogue 2007
miR-128	Apoptosis				
miR-199a	Oncogene activation	Up	Serum	PFOA	Wang J et al. 2012
miR-21	Fatty acid biosynthesis, apoptosis	Up	Blood samples	Arsenic	Kong et al. 2012
miR-26b	Wnt, p53, autophagy, TGF-β				
Let-7a	Cell proliferation, angiogenesis	Up	Cord blood	Arsenic	Rager et al. 2014
miR-16	p53, cell cycle, JAK/STAT				
miR-17	DNA damage response				
miR-20a	Angiogenesis				
miR-20b	Hypoxia				
miR-26b	Wnt, p53, autophagy, TGF-β				
miR-96	Several unrelated targets				
miR-98	Apoptosis				
miR-107	Targets *Notch2*				
miR-126	Angiogenesis				
miR-195	Tumor suppressomiR				
miR-454	Unknown				
miR-24	OncomiR	Down	Plasma	PAH	Deng et al. 2014
miR-27a	Apoptosis, ERα				
miR-28	Apoptosis				
miR-142	Immunology				
miR-150	Hematopoeiesis	Up	Plasma	PAH	Deng et al. 2014
Abbreviations: OncomiR, miR with oncogenic properties; PAH, polycyclic aromatic hydrocarbon; suppressomiR, tumor suppressor miR.					

## Conclusions

miRNAs are omnipresent in the genome and are important regulators of gene expression in response to intracellular as well as environmental cues. In this review, we examined the response of the miRNA machinery to personal and environmental exposures, including air pollution, cigarette smoking, and chemicals such as endocrine disruptors. miRNAs have been proposed as biomarkers for disease; however, the literature also reveals their potential to be used as biomarkers of environmental exposure.

In different studies on the same environmental pollutant, overall the identified miRNAs showed similar patterns of expression regulation. In studies where smoking-induced changes were investigated, the general observation was a down-regulation of expression. For example, miR-125b was down-regulated in response to cigarette smoke in both primary human bronchial epithelial cells (Schembri et al. 2009) and mouse lung tissue (Izzotti et al. 2009). However, when unique miRNAs had altered expression patterns in response to different environmental exposures, their direction of regulation could be the same (10/25 miRNAs) or the opposite (15/25 miRNAs; 60%). The different exposures we discussed here have their own unique health effects, so one would not expect them to have the same effect on the miRNA machinery. However, there is sometimes a discrepancy when looking at the same exposure indicator; for example, in response to smoking, miR-21 has been reported to be up-regulated in some studies and down-regulated in others (Table 4). Part of the discrepancy can be explained by the different exposure models that were used.

In general, different *in vitro* studies show little overlap, potentially because of the complex miRNA–mRNA networks that underlie the observations and the differences in exposure used across studies. In studies of the same environmental pollutant *in vitro*, *in vivo*, or in humans, identified miRNAs were quite distinct (Figures 3–5). This can be explained in part by the observation that animal models do not always reflect genomic responses that occur in humans (Seok et al. 2013). Discrepancy between different studies might also stem from differences in exposure duration. For example, in a study in rats, the duration of exposure uniquely influenced expression patterns of the individual miRNAs (Izzotti et al. 2011).

Human epidemiological studies are necessary to observe exposure-related effects on miRNAs. Understanding the exposome requires putting together pieces of a complex puzzle. Epidemiological studies need input from experimental studies to identify good candidate biomarkers, and results from epidemiological studies often need follow-up by experimental studies to investigate mechanisms of action and to study tissue dependency of effects because human studies are most often performed in easily accessible tissues such as blood and saliva as a surrogate for the actual target tissues.

Currently, epidemiological studies on microRNA often involve free or exosomal miRNAs present in saliva or other body fluids. However, it is not clear whether these observed miRNA changes are a true reflection of the body’s response and can really predict health effects. In blood, miRNAs within the exosomes have been shown to overlap with cellular miRNA profiles: Cheng et al. (2014) observed that exosomes derived from blood were enriched for miRNAs and that miRNA profiles between blood cells and the cell-free exosomal fraction showed important overlap.

Because miRNAs can regulate mRNA expression in both a negative manner and a positive manner (Vasudevan et al. 2007) and because many miRNAs can bind the same mRNA (Saetrom et al. 2007), it is difficult to draw conclusions from miRNA studies without infomation on the concurrent mRNA(s) expression pattern. However, this information is rare in current reports on epidemiological studies of miRNAs. The findings of this review underscore the complex networks that are built by miRNAs and the mRNAs they regulate because one miRNA can influence many mRNAs according to the timing and pattern of expression.

Many of the reviewed studies used large-scale microarray profiling, but follow-up and validation with more quantitative approaches often lags behind. This delay is understandable because of the cost and labor intensity inherent to these techniques; however, it is important to confirm the miRNAs that are responsive to environmental exposures.

Researchers are currently publishing extensive lists of miRNAs that are responsive to environmental exposures and showing their utility as biomarkers of effect. Future research should focus on identifying the molecular mechanism behind miRNA expression changes in response to exposure to determine whether the changes in miRNA expression are merely a symptom of the (patho)physiological processes the organism undergoes after exposure, or whether miRNAs are the drivers responsible for these changes. Izzotti and Pulliero (2014) recently reviewed the putative mechanisms of action behind miRNAs’ response to environmental exposure. However, the effect of the identified miRNAs on putative mRNA targets should also be studied to determine whether the change in miRNA expression has functional consequences and which mRNAs are true miRNA targets under the given circumstances.

At present, little is known about whether environmental exposures induce long-term changes in human miRNA expression or whether these have a transient character. To address this problem, more longitudinal studies should be conducted to examine the long-term effects of exposure. Results from animal studies suggest that miRNA expression changes in response to formaldehyde exposure are transient and revert to normal levels after recovery from exposure (Rager et al. 2014), but Izzotti et al. (2011) reported that miRNA profiles in target organs did not recover 1 week after cessation of long-term cigarette smoke exposure. In a study in humans, Takahashi et al. (2013) observed that miRNA expression profiles of individuals who quit smoking resembled those of nonsmokers.

Follow-up in future generations is necessary to determine the heritability of the miRNA expression changes. It would be very interesting to examine the effect of *in utero* environmental exposures on development of disease in later life and the role miRNAs play in inducing these health effects. Furthermore, long-term longitudinal studies would allow us to distinguish between cause and effect of miRNA expression and environmental exposure, and would also allow us to estimate the contribution of miRNAs to disease development. Studies have shown that miRNAs can be used as biomarkers of disease as well as biomarkers for environmental exposure and that miRNAs hold great potential to explain disease etiology.
